# Combination of Wearable Multi-Biosensor Platform and Resonance Frequency Training for Stress Management of the Unemployed Population

**DOI:** 10.3390/s121013225

**Published:** 2012-09-27

**Authors:** Wanqing Wu, Yeongjoon Gil, Jungtae Lee

**Affiliations:** Graduate School of Computer Science and Engineering, Pusan National University, Busan 609-735, Korea; E-Mail: kyzoon@pusan.ac.kr

**Keywords:** multi-biosensor platform, wearable biofeedback system, resonance frequency training, heart rate variability, unemployment, stress management

## Abstract

Currently considerable research is being directed toward developing methodologies for controlling emotion or releasing stress. An applied branch of the basic field of psychophysiology, known as biofeedback, has been developed to fulfill clinical and non-clinical needs related to such control. Wearable medical devices have permitted unobtrusive monitoring of vital signs and emerging biofeedback services in a pervasive manner. With the global recession, unemployment has become one of the most serious social problems; therefore, the combination of biofeedback techniques with wearable technology for stress management of unemployed population is undoubtedly meaningful. This article describes a wearable biofeedback system based on combining integrated multi-biosensor platform with resonance frequency training (RFT) biofeedback strategy for stress management of unemployed population. Compared to commercial system, *in situ* experiments with multiple subjects indicated that our biofeedback system was discreet, easy to wear, and capable of offering ambulatory RFT biofeedback.Moreover, the comparative studies on the altered autonomic nervous system (ANS) modulation before and after three week RFT biofeedback training was performed in unemployed population with the aid of our wearable biofeedback system. The achieved results suggested that RFT biofeedback in combination with wearable technology was capable of significantly increasingoverall HRV, which indicated by decreasing sympathetic activities, increasing parasympathetic activities, and increasing ANS synchronization. After 3-week RFT-based respiration training, the ANS's regulating function and coping ability of unemployed population have doubled, and tended toward a dynamic balance.

## 1. Introduction

Psychophysiological self-regulation refers to the ability of a person to control affective and cognitive states based on autonomic and central nervous system functioning. This technique utilizes physiological indicators of these states and provides feedback to facilitate the learning of these associations, as well as shows the manner in which their occurrence can be modulated in order to achieve optimal human physiological functions [1]. Current biofeedback research primarily focuses on biofeedback theory and instructions/guidelines. Several heart rate variability (HRV) biofeedback strategies have been developed to effectively increase cardiac variability, such as heart rhythm coherence feedback, oscillatory biofeedback [2], and resonance frequency training (RFT) feedback [3]. On the other hand, some research merely paid attention to the hardware platform of biofeedback apparatus. For example, the MIT Media Lab developed MIThril—A wearable computing platform compatible with different off-the-shelf sensors [4]. Chulsung and Chou developed an Eco platform, which is a self-contained, ultra-wearable, and expandable wireless sensor platform [5]. The University of California, Berkeley, designed an open-source platform [6]. Yang and others from Imperial College, London, presented a bioinspired processing-on-node platform [7]. Previous study also included a body area sensor network (BSAN) platform [8] and a phone-based platform [9]. However, relatively little attention has been paid to the combination of the sophisticated wireless biosensor platform and the advanced biofeedback strategy for self stress management and biofeedback training.

With global challenges, we are constantly suffering from negative emotions such as depression and stress anytime and anywhere, which dramatically impairs our health and performance. Since the global recession began in 2008 until the first quarter of 2012, the unemployment rate rose from 5.0% to 8.2% in the USA, from 7.6% to 10.2% in the EU (Bureau of Labor Statistics, 2011; Eurostat) [10], and from 3.2% to 4.2% in the Republic of Korea. Especially, the youth unemployment rate in the Republic of Korea has been averaging around 8% in 2008 and raised to 10% in 2012 (Statistics Korea). There is substantial evidence that unemployment has a deleterious impact on psychological and physical health. Thus, relative to the employed persons, unemployed people experience an increased prevalence of mental stress, depression, and hypertension, in addition to elevated rates of cardiovascular disease and all-cause mortality [10]. Consequently, biofeedback strategies frequently used to cope with stress include relaxation techniques, promotion of a healthy lifestyle, and cognitive-behavioral therapies.

Traditionally, these strategies require significant professional training, expertise and equipment to administer as well as people, time, and resources, which can be difficult to achieve. To overcome these limits, studies on the integration of wearable computing and mobile devices for biofeedback purposes have received considerable attention from the research community during the last several years [11–20]. The intrinsic requirements of wearable computing, such as light-weight, low energy consumption, high computational performance,environment/activity awareness, fashionable, large memory, network connectivity, and cost, have offered distinct advantages and benefits that outweigh the limitations or difficulties involved in traditional biofeedback methodology. The effectiveness of wearable computing and devices in biofeedback application has also been verified by many researchers. For example, the research conducted by Cipresso *et al.* [11] has showed that it is possible to induce positive or negative affective states using a smartphone. A handheld biofeedback device called Stress Eraser has been designed by Moore [12], which could provide visual and audible cuesto reduce stress level.In the literature [13], Zhang *et al*. introduced a prototype wearable respiration biofeedback platform to guide respiration. Gerasimov *et al.* [15] described a class of wearable data acquisition systems called “extremity-computing devices” for stress monitoring and biofeedback training.

Nevertheless, little research has been devoted to integrating biofeedback training methods into the wearable biofeedback system. Although the wearable computing and devices could provide physiological indicators for individuals, biofeedback training still depends on the instructions ofspecialists. Accordingly, the combination of biofeedback training strategies with wearable biofeedback system for stressmanagement is undoubtedly meaningful. It can not only promote the wide use of the biofeedback techniques to enhance health in real life, but also offer a more integrative platform for deeper research on the mechanism of the stress-related biofeedback techniques.

Therefore, the purpose of this study was to implement a wearable biofeedback system, with an integrated application that uses multi-biosensor platform to gather raw physiological data and a ubiquitous smartphone (or notebook and PDA) to process and display real-time physiological information for stress assessment. More important, we also integrated RFT- based HRV biofeedback strategy into the system. Extensive bench and *in situ* experiments with multiple subjects have been performed to compare the performances between our wearable biofeedback system and a commercial system (Laxtha PolyG-A). Finally, comparative studies on the responsiveness of the cardiac autonomic system between the unemployed (33 subjects) and employed (34 subjects) population, and the alterations of the ANS modulation before and after 3-week RFT biofeedback training in unemployed population have also been performed with the help of our wearable biofeedback system. The paper is organized as follows: Section 2 provides some background on physiological measurement and mechanism of biofeedback methodology in this study. Section 3 introduces the architecture and implementation of our wearable biofeedback system. Section 4 describes the system integration and the performances evaluation in comparison with commercial system. Section 5 presents comparative studies on the differences of ANS's regulating function and coping ability between the employed and unemployed population, and the effects of 3-week RFT biofeedback training for unemployed population by virtue of our wearable biofeedback system. Finally, Section 6 presents our conclusions.

## 2. Biofeedback Methodology

### 2.1. Physiological Measurement

Biofeedback is an extensive interdisciplinary technique through which clients train themselves to acquire a set of skills, the learning of which is elaborated through the information given by a biofeedback apparatus. As a health intervention technique, biofeedback is well known to facilitate treatment for a wide variety of disorders with a psychosomatic component, including asthma, cardiovascular disorders, hypertension, cephalopathies, anxiety, and duodenal ulcers, and in many cases, the results obtained have been notably positive. However, relatively little attention is being paid to the portability of biofeedback apparatus, especially for the wearable biofeedback platform. For example, most researches such as HRV biofeedback employed bulky desktop devices and were conducted in advanced laboratory spaces, which inhibited the possibilities of low cost and ubiquitous health intervention services [12].

On another side of the spectrum, technology advantages on microelectronics and computing have led to the exploitation of body sensor networks (BSNs) that monitor client's health status ubiquitously. A BSN is typically equipped with different sensors, such as photoplethysmography (PPG) sensor, electrocardiogram (ECG) sensor, respiration sensor, electromyography (EMG) sensor and noninvasive blood pressure sensor, for on-body/in-body physiological measurements. Consequently, the data acquired from the multiple sensor nodes are relayed wirelessly and in real time to the master node, where the data are processed autonomously. It is evident that the continuous development of BSN platforms could lead to emerging biofeedback apparatus as well as novel biofeedback applications.

Due to the characteristics of complexity, universality, and comprehensiveness, it is difficult to build a universal standard for biofeedback methodology. Therefore, it is meaningful to design an integrated wearable biofeedback platform, which is able to offer unemployed clients advanced biofeedback services needed for affordable healthcare.

### 2.2. Physiological Mechanism

A number of physiological markers of stress have been identified, including electrodermal activity (EDA), heart rate (HR), various indices of HRV, blood pressure (BP), muscle tension, and respiration, among which the HRV is one of the most robust physiological predictor of mental or physical stress. HRV is thought to reflect the heart's ability to adapt to changing circumstances by detecting and quickly responding to unpredictable stimuli. HRV analysis is the ability to assess overall cardiac health and the state of the ANS responsible for regulating cardiac activity. Therefore, it is impossible to evaluate the stress responses and level of unemployed population by using HRV indices.

RFT feedback as a specific biofeedback training strategy is aimed at producing maximal increases in the amplitude of respiratory sinus arrhythmia (RSA) [21]. Based on the resonance properties of the cardiovascular and respiratory system, RFT can promote a homeostatic state in the body, and generate high-amplitude oscillations in autonomic functions by using slow-paced breathing at each individual's resonant frequency (usually approximately 0.1 Hz, 6 breaths/min). This resonant frequency is represented by a lower frequency band in the HRV power spectral density (PSD) and reflects sympathetic and parasympathetic autonomic control and the maximum gain of the baroreflex feedback system [3]. In addition, it is now recognized that the variability (rhythm) of the heart is an indicator of both physiological resiliency and behavioral flexibility, reflecting a person's capacity to adapt to stress and environmental demands. There is abundant evidence that positive emotions affect the activity of the body's physiological systems in profound ways. For instance, studies have shown that positive emotional states speed the recovery of the cardiovascular system from the after effects of negative emotions [22], alter frontal brain asymmetry and increase immunity [23]. Finally, the use of practical techniques that teach people how to self-induce and sustain positive emotions and attitudes for longer periods has been shown to produce positive health outcomes. The heart rhythms associated with positive emotions, such as appreciation, are clearly more coherent (organized as a stable pattern of repeating sine waves) than those generated during a negative emotional experience such as frustration. The consistent and pervasive influence of the heart's rhythmic patterns on the brain and body not only affect our physical health but also significantly influence perceptual processing, emotional experience, and intentional behavior [24].

Our concept of the biofeedback methodology is based on such theoretical basis of physiological responses, the relationships among stress, HRV and respiration, and the construction of a new integrated wearable biofeedback platform. It is possible to create an easier, more accurate, and more cost effective multi-scenario (regular, ambulatory) physiological recording (ECG, PPG, respiration, *etc.*). Then, training jobless subjects to take appropriate action to control the tonic level of various physiological functions becomes more straightforward and much simpler.

## 3. System Architecture

**Figure 1.**
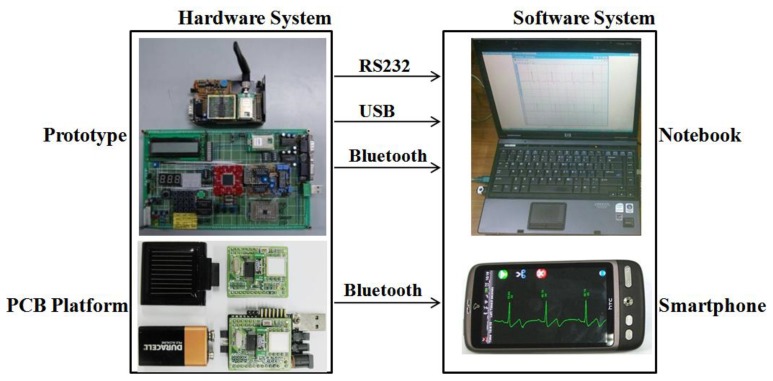
The system architecture.

In this study, we designed a prototype biofeedback system and corresponding wearable sensor platform with low-cost, off-the-shelf components, aiming at performance and ergonomics typical of consumer market products. The platform was designed to include modular hardware architecture for diverse sensor integration, signal conditioning, power management, optional peripheral circuits, and wireless communications, as well as a modular software design to allow easy and optimized integration of different digital signal processing algorithms, characteristic parameter analysis and extraction, communication protocols, and biofeedback algorithms.The system architecture was designed to potentially include a network of sensing nodes and is capable of driving different actuators as biofeedback generators (e.g., audio, tactile, and visual). In the current release, it is basically composed of two main parts:a hardware system and a software system (Figure 1).

### 3.1. Hardware Architecture

Given that the primary purpose of the multi-biosensor platform is to help the user to self-correct and regulate his/her physiological functions both in static and dynamic conditions, the major requirement for the multi-biosensor platform is the ability to provide the user with all the necessary tools to be independent from subjective and objective interventions. Specific requirements of the system taken into account for the design are (i) wearability and unobtrusiveness, requiring light and small-sized components; (ii) low-cost (affordable for the consumer market); (iii) low-power consumption (supporting daily continuous use); (iv) flexibility and easy integration with other sensors and actuators; (v) easy maintenance and update of components; and (vi) mobility for ubiquitous use.

**Figure 2.**
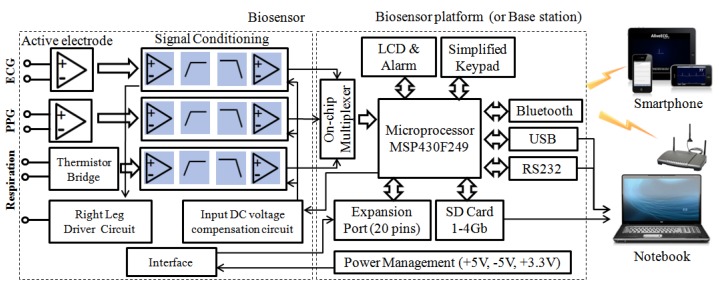
The hardware architecture.

Based on such considerations, thecurrent release of our multi-biosensor platform is composed of four components: one or more sensor nodes, a biosensor platform, a base station node, and power solutions (power adapter interface and portable battery), which is illustrated in Figure 2. In this study, we focused on the ECG and respiration sensor because our application of the biofeedback algorithm combined the heart rhythm pattern with resonant frequency respiration training.

#### 3.1.1. ECG/PPG/ Respiration Sensor

Physiological signals are usually weak and easily obscured by undesired noise (ECG signals typically have amplitude in the range of 1–3 mV), therefore, both amplification and filtering are required for further signal processing. Moreover, human skin typically provides source impedance on the order of 1–5 MΩ, consequently amplifiers must match the source impedance or have greater input impedance than the source skin impedance to acquire surface physiological signals. To avoid 60 Hz power line noise, a narrow band notch filter is also needed for signal processing. For these reasons, a relatively high common mode rejection ratio (CMRR) is necessary to reject common mode signals. Thus, a high input impedance, high CMRR, and moderately high gain instrumentation amplifier will be a good choice as a differential amplifier for the ECG, PPG and respiration signal conditioning circuit. In this work, the instrumentation amplifier chip INA128AIM [25] is selected, which is a low-power, general-purpose instrumentation amplifier with low input bias current. It also features a high CMRR of 120 dB and a differential input impedance of 10 GΩ∥2 pF.

In order to acquire the most useful ECG and PPG band, the cutoff frequencies of the low-pass filter (LPF) and the high-pass filter (HPF) are set to 0.5–40 Hz for ECG signals and 0.1–20 Hz for PPG signals, respectively. The output signal of the instrumentation amplifier is transferred to a 5th-order Bessel LPF with 0 dB gains. After the LPF, the signal is then filtered by a 2nd-order Butterworth HPF to attenuate the low frequency signal with 0 dB gains. Then, the signal is filtered by a 60 Hz notch filter to reject the power line noise. Finally, the signal is amplified by a 2nd invert amplifier with 40 dB gains for ECG signal and 20 dB gains for PPG signal. Specifically, we used active electrodes (composed of voltage followers) instead of conventional Ag-AgCl electrodes in order to overcome the imbalanced electrode-skin impedance, which results in the noise's transformation from common mode to differential mode. Further, the right leg driver is implemented in the ECG sensor to reject common mode noise in the body and resist the 60 Hz noise.

**Figure 3.**
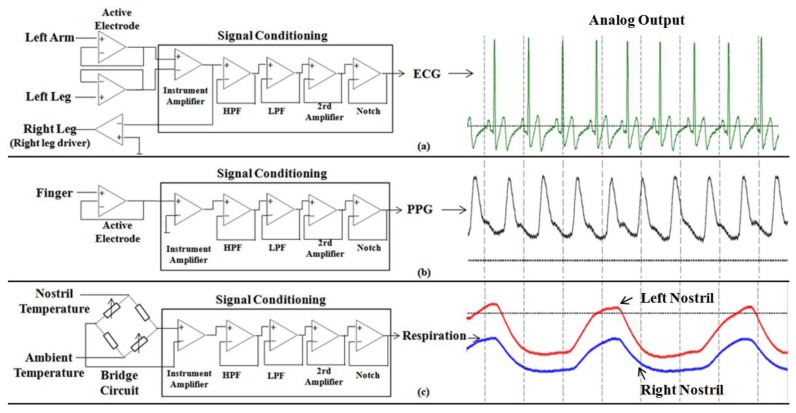
The structure of biosensor and corresponding analogy output; (**a**) ECG sensor; (**b**) PPG sensor; (**c**) Respiration sensor.

The respiration sensor used two thermistors to detect changed in the breath temperature between ambient temperature (inhalation) and lung temperature (exhalation). A thermistor placed in front of a nostril is configured in the form of a bridge, with calibration resistors (thermistor) used as a reference (to detect ambient temperature). The operation amplifier LM324 [26] was used to amplify the signal, and the similar methodology for signal conditioning, as described in the ECG / PPG sensor, has also been applied for conditioning the respiration signal with distinct parameter specifications (LPF, 0.1 Hz; HFP, 10 Hz; 2rd Amplify, 10 dB gains). The specifications of sensor's amplifier and filter part are listed in Table 1, and the structures of ECG sensor, PPG sensor and respiration sensor are illustrated in Figure 3.

#### 3.1.2. Multi-Biosensor Platform

A low power microcontroller (Texas Instruments MSP430F249 [27]) for data acquisition and processing), a Bluetooth module (Firmtech FB155BC [28]) for wireless communication, a 20-pin expansion port, a power regulator/power adapter interface, a USB/RS232 communication interface (allowing direct connection to computer or smart devices), and the affiliated discrete components were integrated into the multi-biosensor platform.

TI's MSP430F249 was selected because it is an ultralow-power 16-bit reduced instruction set computer (RISC) processor within the TI MSP430 family, and it provides the best combined performance in power consumption and flexible clock subsystem. In the multi-biosensor platform, the sensor nodes, the Bluetooth module, the USB/RS232 interface, and some optional components (SD card, LCD, simplified keypad, *etc.*) were all controlled by the microcontroller through relevant ports (universal I/O, SPI, UASRT, *etc.*).The embedded FB155BC Bluetooth module was designed by Firmtech Co. Ltd (Korea) and was used to realize stable wireless communication, even in extremely noisy environments (by adopting the FHSS technique), owing to its low power consumption, high reliability, and low cost.

The expansion port has 20 pins, including eight for analog signals, four for generic-purpose digital input/output (I/O), three for SPIs, four for digital and analog power supplies, and one for battery voltage. This port enables the multi-biosensor platform board to connect with a variety of biosensor interfaces (PPG; Pulse Oximeter Oxygen Saturation, SpO_2_; Electroencephalogram, EEG; *etc.*) and battery or charger boards. Multi-biosensor platform can be connected to personal computer or smart phone directly. It can also serve as the master node within the body sensor network (BSN).

#### 3.1.3. Base Station

The base station node also includes an MSP430F249 microprocessor and an FB155BC module, which are identical to the multi-biosensor platform. The base station board provides RS232 and universal serial bus (USB) connections, four LEDs, and three keys for user-device interactive purposes. Once connected with a personal computer (notebook) or PDA device, the base station board serves as the master node within the body sensor network. In this study, one base station serves one subject with multi-sensor nodes (N slave node: one master node). If a multi-biosensor platform exists, the base station node is also in charge of receiving data packets from the different biosensor platforms and transferring the data to a notebook (or PDA) correctly, with stream control mechanism.

#### 3.1.4. Power Solutions

In the multi-biosensor platform, we have integrated an abundance of power interfaces for different types of adapters (3.3 V, 5 V, 9 V, and 12 V) in static scenarios. Most importantly, we have also provided three portable power solutions: a 9V battery with a capacity of 350 mAh, button cell battery (3 V) with a capacity of 250 mAh, and a rechargeable portable solar battery (3.7 V) with a capacity of 350 mAh, which allow larger flexibility and adaptability for the multi-biosensor platform in dynamic scenarios.

### 3.2. Software Architecture

The software architecture has been designed to best accomplish real-time data processing, storage, and transmission; I/O synchronization; biosignal extraction, analysis, and display; physiological parameters detection and assessment; trouble-free integration of further biofeedback algorithms; independence from sensor node(s) characteristics; and setup. Such features have been combined with a friendly graphical user interface (GUI) in notebooks and smartphones for non-expert users and allow the caregiver or supervisor to select appropriate biofeedback strategies and user-specific options.

**Figure 4.**
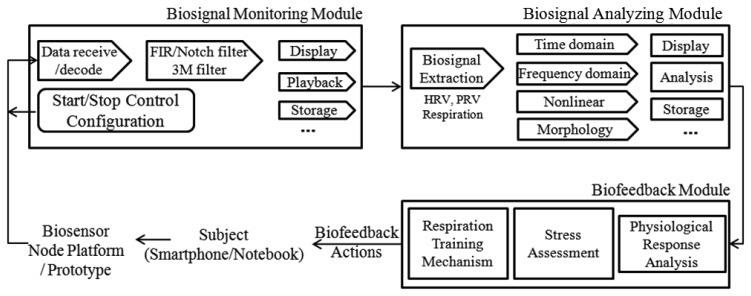
The software architecture.

The functioning principles of the software system and its architecture are illustrated in Figure 4. In summary, the main building blocks of the software architecture consist of three components: a biosignal monitoring module, a biosignal analyzing module, and a biofeedback module.

#### 3.2.1. Biosignal Monitoring Module

The monitoring module is in charge of enabling communication between the hardware platform and the smartphone or notebook, extracting meaningful data (payload) from the packets received, preprocessing the raw biosignal (ECG, PPG and respiration). With a friendly GUI, monitoring module also used to configure related parameters (communication, measurement, biofeedback), display and playback the biosignal waveform, save real-time raw data and analysis results, and handle basic functionalities (e.g., start and stop measurement or the application).

As previously mentioned, physiological signals are usually weak and easilycorrupted by various kinds of noise (power line interference, electrode contact noise, baseline drift, instrumentation noise, motion artifacts, electrosurgical noise, and other less significant noise sources), which cannot be filtered completely by the hardware platform. Therefore, a digital 60 Hz notch filter for minimizing the power line interference, a finite impulse response (FIR) band-pass filter for correcting baseline wander, a multi-scale mathematical morphology (3M) filter [29] for eliminating electrode contact noise and motion artifacts, and a differential operation method (DOM) for smoothing and normalizing have been integrated into the monitoring module.

**Figure 5.**
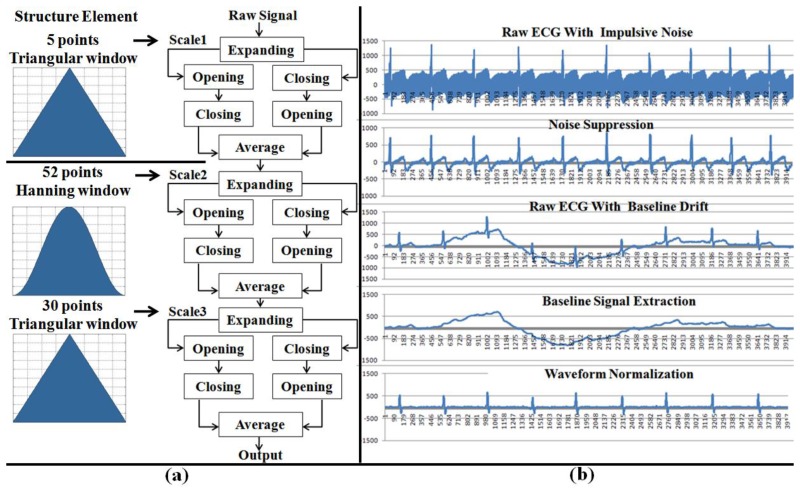
The structure of 3M filter and corresponding filter effects. (**a**) the structure element in different scales (scale 1 for impulsive noise suppression; scale 2 and 3 for waveform normalization); (**b**) filter effects during the stage of impulsive noise suppression and waveform normalization.

The 3M filter, based on set operations, provides a way to analyze signals using nonlinear signal processing operators that incorporate the geometry information of the signal. The shape information of the signal is extracted by using a structure element to operate on the signal. In this module, the 3M filter is used to eliminate baseline drift and reduce interference noises (motion artifacts, muscle contraction), which includes two steps: impulsive noise suppression and waveform normalization (baseline correction). The structure of the 3M filter and the filtered effects are illustrated in Figure 5.

#### 3.2.2. Biosignal Analyzing Module

The analyzing module is responsible for extracting bioinformation from raw data and for calculating the corresponding characteristic parameters (HRV, PRV, rhythm pattern and inter-breathing interval) of the biosignal for monitoring and biofeedback purposes. In this study, HRV, heart rhythm pattern, and breathing parameters have been taken into consideration.

**Figure 6.**
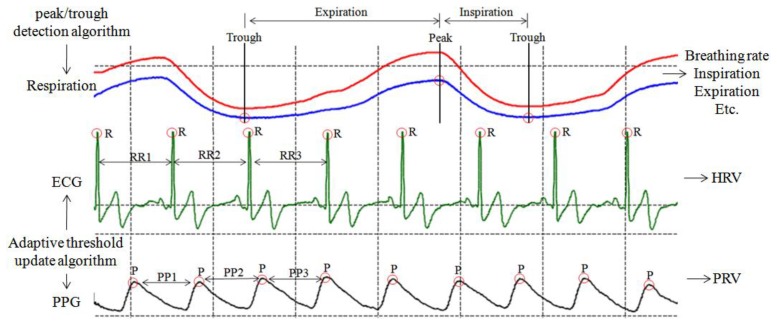
The characteristic values of ECG, PPG and respiration signal. HRV and PRV signal are extracted by adaptive threshold update algorithm, and respiration parameters are calculated by peak/trough detection algorithm.

In order to extracted the HRV (or PRV) series and heart rate (or pulse rate) rhythm pattern from raw ECG (or PPG) signal (Figure 6) for stress assessment and biofeedback application, an adaptive threshold update algorithm, which is easy to implement on a simple, real-time device developed by our laboratory in a previous study, has been adopted to extract RR (or PP) interval series for HRV (or PRV) analysis, with 99.3% detection rate [30].

In order to obtain respiration parameters (Figure 6), the peak/trough detection algorithm [30] is also designed to identify the critical parts of each breath in a respiration signal recorded by the respiration sensor. Once these critical points (peak and trough) have been identified, statistics such as breathing rate and relative timing of inspiration and expiration within respiratory cycle can be calculated (Table 2).

As shown in Table 2, the calculated time domain (SDNN, rMSSD) and frequency domain (Tower Power, LF norm, HF norm, LF/HF ratio) of HRV (or PRV) are obtained according to the standards of measurement, proposed by the Task Force of the European Society of Cardiology and the North American Society of Pacing and Electrophysiology [31], which describes the details of physiological correlates of HRV (or PRV) and calculation methods. Furthermore, heart rate (or pulse rate) rhythm pattern analysis is performed by the morphological inspection and quantitative method (coherence ratio, CR) [24] simultaneously, which provides an accurate measure of coherence that allows for the nonlinear nature of the HRV waveform over time.

#### 3.2.3. Biofeedback Module

In this study, the biofeedback module was built on the physiological associations between stress and HRV indices [31], and the theoretical basis of HRV biofeedback strategies for stress reduction and ANS modulation through resonance frequency respiration training.

As we mentioned in Section 2.2, HRV is one of the most robust physiological predictors of mental or physical stress. The temporal measure SDNN which reflects overall ANS activity is the most representative parameter of HRV indices, and the clinical meaning of decrease in SDNN indicates weakened ANS ability to maintain homeostasis against internal/external environmental challenges, lowered coping ability to various emotional/physical stressors and general health weakness. Therefore, SDNN was selected to assess the stress level. Furthermore, the spectral measure LF/HF ratio was used to help quantify the overall balance between the sympathetic and parasympathetic systems. If the value of LF/HF ratio lower than 0.5 indicates impaired modulation of sympathetic nervous system (SNS) induced by chronic stress, whereas the value of LF/HF ratio higher than 2 represents suppression of parasympathetic nervous system (PNS) caused by acute stress [31]. Table 3 illustrated the corresponding relation between the mean values of HRV measures and stress level.

**Figure 7.**
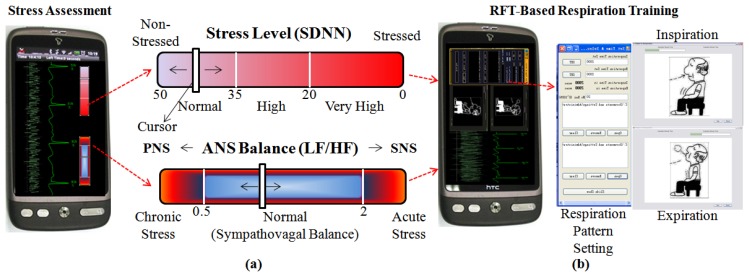
(**a**) The GUI for stress assessment; (**b**) the GUI for RFT-based respiration training.

According tothis corresponding relationbetween stress and HRV, we have designed a GUI for self-evaluation of stress level. As shown in Figure 7(a), the upper one is stress level reflecting the SDNN in time domain. If the white cursor goes to right side, it means the pressure on the heart gets higher. An individual with higher stress level is more likely to have psychosomatic disorders.The lower one is the stress status that comes from the balance between SNS and PNS (LF/HF). Although the balance between them is not the sole factor associated with an individual's stress status, this gives some useful clues. For instance, the chronic stress induced by long illness or psychological diseases moves the cursor to the left side, while the acute stress caused by feelings of anger or tension moves the cursor to the right side. Also, we designed a GUI for RFT-based respiration training Figure 7(b), which allows the individual to easily and conveniently learn RFT-based respiratory skills for stress reduction and biofeedback training.

**Figure 8.**
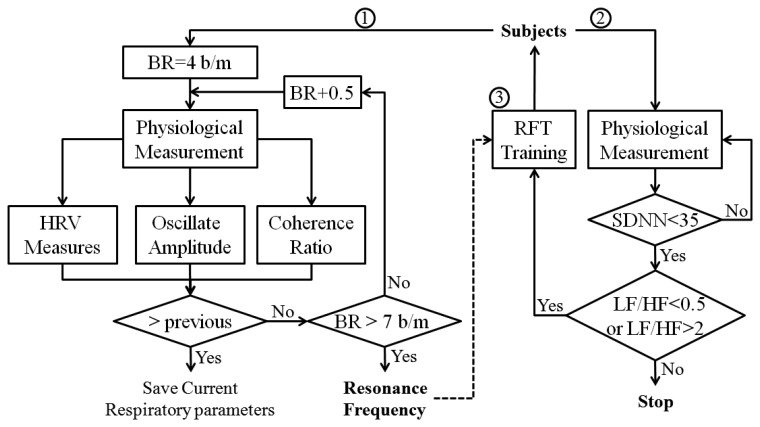
The flowchart of biofeedback algorithm; Step 1, find individual's resonant respiratory frequency; Step 2, stress assessment; Step 3, RFT-based respiration training.

Current research suggests that each individual has a “resonant frequency” at which the HRV is the largest, and this resonant frequency can be measured with biofeedback instruments. While it is not uniform or ideal for everybody, this resonant frequency tends to occur in a relaxed mental state, with positive emotional tone, breathing diaphragmatically and smoothly at a rate of approximately 4–7 breaths/min (includes 99% of population, 5–6 breaths/min in approximately 50% of population). Therefore, the biofeedback algorithm designed for stress management is composed of three steps. In the first step, cyclic measurements were performed at different respiratory frequencies (from 4–7 breaths/min, in increments of 0.5), and the resonant respiratory frequency was recorded when the maximum gain of HRV was achieved. Then, the subject is asked to practice breathing at his/her resonant respiratory frequency with the help of the biosignal monitoring module, while trying to keep the depth of respiration approximately constant. In the second step, assess the stress level according to the correspondence relation between stress and HRV indices (Table 3). In the third step, the subject utilizes the resonant frequency respiration training for stress reduction. The flowchart of the biofeedback algorithm is illustrated in Figure 8.

## 4. System Integration and Evaluation

**Figure 9.**
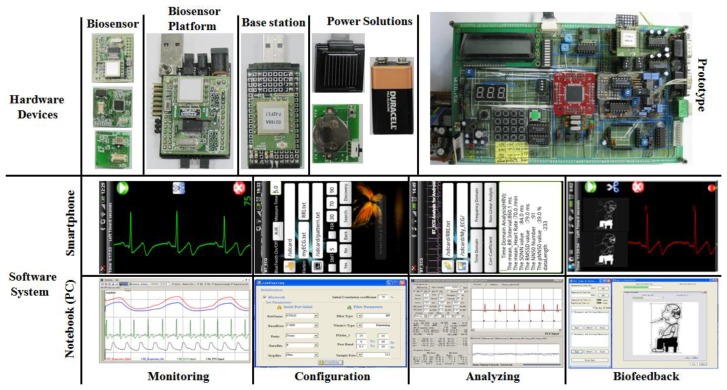
The implementations of the hardware devices (prototype and wearable) and software system (smartphone and notebook).

**Figure 10.**
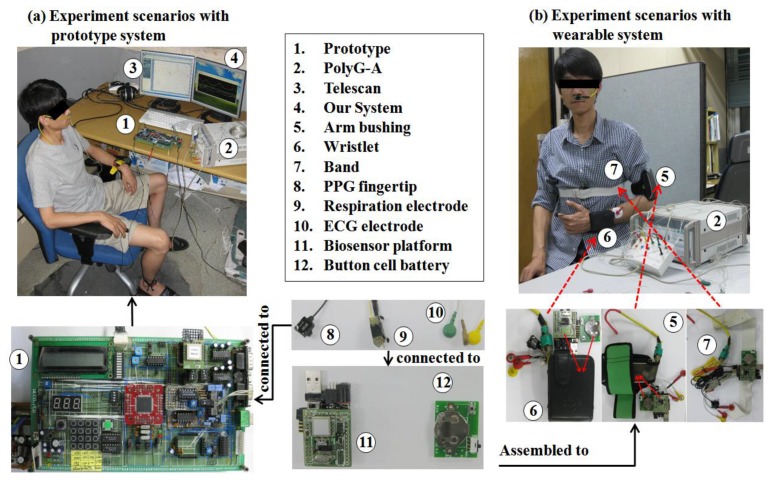
(**a**) The experimental scenarios with prototype system (desktop); (**b**) the experiment scenarios with wearable biofeedback device.

Figure 9 illustrates the implementations of the hardware and software systems. The wearable biofeedback device (ambulatory) was assembled using the aforementioned multi-biosensor platform and associated sensors and electrodes, which is illustrated in detail in Figure 10. We provided three integration schemes: this wearable biofeedback platform could be used as a wristlet or an arm-bushing, inside of which there were a group of sensor nodes, a multi-biosensor platform, and a battery board with a button cell and relevant electrodes. Also, it could be sewed on the waist band or thoracic belt as an optional scheme. The subjects could choose the most convenient scheme to wear this biofeedback system.In our current design, the sensor nodes included an ECG sensor, a respiration sensor, and a PPG sensor (optional). During the experiment, the wearable device, base station node, smartphone and notebook (or PDA) formed a body sensor network (BSN). Figure 9 also illustrates the experiment scenarios with prototype system.

In order to evaluate the performances of this system, *in situ* dynamic respiration experiments were conducted with multiple subjects in an air-conditioned room. Subjects sat upright while wearing the biofeedback device on the wrist or elbow. A commercial device named “PolyG-A” and self-contained software named “Telescan” from LAXTHA Inc. were used for performance comparisons under the same conditions.

**Figure 11.**
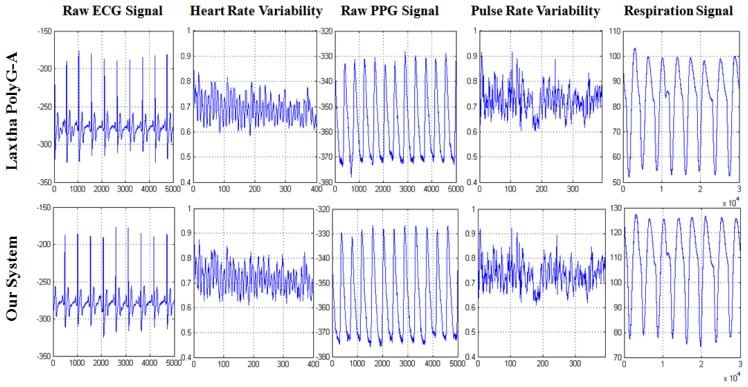
The performances comparison of LAXTHA PolyG-A system and our biofeedback system; raw ECG, PPG and respiration signal are recorded simultaneously by PolyG-A device and our device; HRV and PRV curve are also extracted simultaneously by Telescan and our software system.

During the experiment, PolyG-A system and our biofeedback system (prototype and wearable system) shared the same ground and measure electrodes (ECG, Lead II, PPG, forefinger; respiration, left nostril) with the same sampling rate of 512 Hz. The ECG, PPG and respiration signal were recorded simultaneously by PolyG-A and our system. Figure 11 illustrates a segment of the acquired ECG, PPG and respiration signal,which indicated that ECG, PPG and respiration signal were successfully detected by our device, and have similar waveform with PolyG-A. Furthermore, HRV curves and PRV curves (Figure 11) also were extracted simultaneously by using Telescan and our system; the results exhibited high similarities, which indicated that HRV and PRV signal were correctly extracted by our software system, and were capable of being used for stress assessment and biofeedback application.

Low power consumption is the most significant challenge for any wireless sensor network. In this study, we also measured the battery life of the aforementioned three portable power solutions during actual biosignal monitoring in this preliminary evaluation: A 9 V battery with capacity of 350 mAh, a portable solar battery (3.7 V) with capacity of 350 mAh, and a button cell (3 V) capacity of 250 mAh. The biosignal platform with ECG and respiration sensor was in 100% active duty cycle in the experiment. As the sampling rate is 512 Hz and one sample length is 2 bytes, the data rate for transmission of 2 channels was 64 kbps (512 × 2 × 8 × 8). The biosignal platform can work about 10 h with a 9 V battery, 5 h with a solar battery and 3.5 h with a button cell, respectively, for real-time ECG and respiration monitoring under the above conditions.

## 5. Experiments

### 5.1. Participants

The sample consisted of 67 participants (52 men and 15 women) between the ages of 20 and 45 years. 33 participants were unemployed (drawing unemployment insurance) and 34 were employed. Unemployed participants were recruited from the Labor Office branch in the city of Busan (Korea); the employed participants (control group) were recruited from internet advertisements and the distribution of flyers. We excluded those participants that were pregnant, diabetic, had a history of stroke, heart attack or cardiac disease, reported mental illness, had drug abuse or dependence in past, were on cardioactive medication such as beta blockers, had blood pressure above 150/90 mmHg, or did not feel well at the time of testing.

Furthermore, we used twenty two items in a simplified version of the original Stress Response Index (SRI) questionnaires [32] to assess the stress level of subjects before physiological measurement and RFT biofeedback training. Each question in the SRI questionnaire was scored in a Likert-type format: “Not at all” (0 score), “Somewhat” (1 score), “Moderately” (2 scores), “Very much” (3 scores), or “Absolutely” (4 scores). If the total SRI score of the simplified version SRI is over 56, the stress level is interpreted as severe state. According to this criterion, we also excluded those participants in the employed group whose SRI score were over 56 (which indicated serious work-related stress), and those participants in unemployed group who's SRI score were lower than 56 which indicated low or normal stress state [33].

This study has been approved by the Institutional Review Board (IRB) of Pusan National University Hospital. Informed written consent was provided by all participants before completing questionnaires (SRI) or undergoing psycho-physiological assessment.

### 5.2. Procedures

In order to validate the effectiveness of RFT-based HRV biofeedback for stress management of unemployed population with the help of our wearable biofeedback system, the procedures basically included two stages.

In the first stage (*in situ* experiment), Lead II ECG signals, forefinger PPG and two nostril respiration signal were recorded simultaneously by our wearable biofeedback systemwith regular ECG, PPG (optional) and respiratory electrodes as shown in Figure 9(b) for each subject in both groups (unemployed group and employed group), and were telemetrically transferred to a notebook or smartphone. All subjects were tested under standard conditions in our laboratory between 1:00 and 5:00 p.m. at a room temperature of 22–26 °C after abstaining from smoking and coffee consumption for 12 h before participation in the experiment. Regular ECG, PPG (optional) and respiratory electrodes were attached to the participants comfortably.

The recordings collected from employed group rest state were regarded as baseline (BL) dataset. And the recordings obtained from unemployed group at rest state were used to assess the psychophysiological states of the unemployed subjects (pre-RFT).

In the second stage (ambulatory experiment), the subjects of the unemployed group were also asked to wear the wearable device comfortably with the same settings described in first stagefor ubiquitous physiological measurement and RFT biofeedback training. The subjects received three sessions' training (one session per week):

- In the first session, the participants were taught how to produce maximal increases in the amplitude of respiratory sinus arrhythmia (RSA) while breaths at specific respiratory frequencies, ranging from 4 to 7 breaths / min, and keeping depth of respiration approximately constant (as shown in Figure 8, step 1).
- In the second session, the primary task was to monitor progress, measure the HRV parameters, and correct errors in technique. An incorporation of a combination of mental and physical stressor (as shown in Figure 8, steps 2, 3) during this session was suggested, such stressors help the trainee gain proficiency in controlling performance pressure and autonomic activity.
- In the third session, the participants were also asked to practice breathing at his/her resonance frequency for two 20-min sessions per day using our biofeedback system in daily life (home or living environment).

After this 3-week RFT training procedure, HRV indices and respiration measures at rest state were collected from all the subjects in unemployed group, which used to assess the effects of 3-week RFT training (post-RFT).

### 5.3. Parameters and Statistical Analyses

HRV frequency domain analysis included HRV LF (nu), HRV HF (nu) and LF/HF ratio (%), as well as total spectral variability (TP, ms2). Time-domain measures included the SDNN (ms), rMSSD (ms). SDNN and TP were regarded as overall performances of ANS. HRV HF norm and rMSSD were regarded as indices of parasympathetic control; HRV LF norm was regarded as indices of sympathetic control, and LF/HF ratio was used to evaluate the sympatho-vagal balance [31]. Furthermore, the coherence ratio [24] which reflects the degree of ANS synchronization was also taken into consideration. Respiration parameters included breathing rate (BR, b/m), inspiration time (insp. time) and expiration time (exp. time). All of them were used to check the respiratory manipulation, and assess the alterations of respiratory pattern in unemployed population during different experiment sessions.

Data were presented as mean ±standard deviation (SD). Pairwise comparisons were made to compare the difference between the BL and pre-RFT, and the pre-RFT and post-RFT by using the Student's paired t-test (probability values less than 0.05 were considered statistically significant). Differences of HRV indices and respiration measures in different groups (BL, pre-RFT and post-RFT) were also assessed by using one-way analysis of variance (one-way ANOVA) with Bonferroni correction in group comparisons, which was performed to evaluate the effects of the RFT-based biofeedback training on the ANS's regulating function and coping ability, and the alterations of respiratory pattern in unemployed population. Because the Bonferroni correction was used to avoid errors from multiple tests, differences at P < 0.05/3 (≈0.017) were accepted as statistically significant (SPSS v17.0, Chicago, IL, USA).

### 5.4. Results and Discussion

#### 5.4.1. Respiratory Pattern Analyses

In Table 4, the mean values of breathing rate, expiration time, and inspiration time are listed. As expected, all of them were differed significantly between pre-RFT and post-RFT. The breathing rate during the pre-RFT condition in the unemployed group was markedly higher than BL (12.2 ± 0.6 *vs.* 19.3 ± 0.9, p < 0.001) and post-RFT (13.5 ± 1.3 *vs.* 19.3 ± 0.9, p < 0.01) conditions, and no significant difference has been observed between BL and post-RFT. During the pre-RFT condition, mean expiration time was decreased significantly in comparison with BL (p < 0.001) and post-RFT (p < 0.01) conditions, whereas mean inspiration time was increased obviously compared to BL (p < 0.05) and post-RFT (p < 0.05) conditions. Repeated measures ANOVA test also showed that 3-week RFT biofeedback training with our wearable biofeedback system altered the respiratory pattern significantly with decreased breathing rate (F(1, 33) = 19.365, p < 0.001), prolonged expiration time (F(1, 33) = 15.886, p < 0.001) and shortened inspiration time (F(1, 33) = 6.067, p = 0.012).

#### 5.4.2. HRV Analyses (Heart Rhythm Pattern, Time Domain and Frequency Domain)

**Figure 12.**
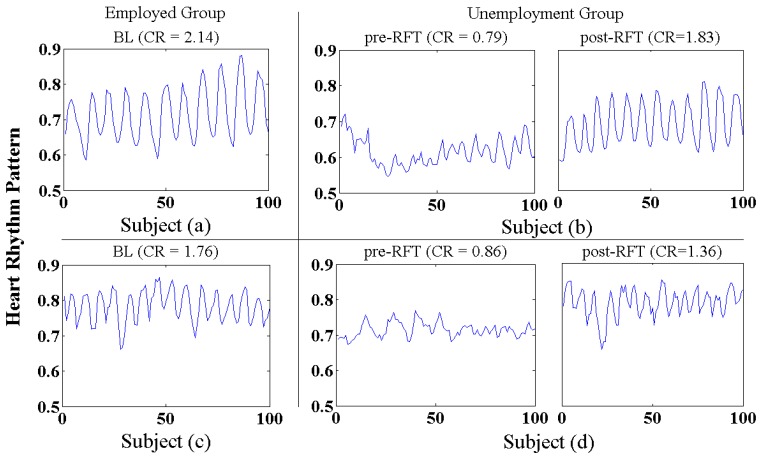
Representative samples of heart rhythm pattern in the BL, pre-RFT and post-RFT conditions; subject (**a**,**c**) derived from employed group in the BL condition; subject (**b**) derived from unemployed group in the pre- and post-RFT conditions; subject (**d**) derived from unemployed group in the pre- and post-RFT condition.

Figure 12 shows representative samples of heart rhythm patterns derived from the employed (BL) and unemployed group (pre- and post-RFT), it can clearly be seen that morphological differences existed between the BL and pre-RFT conditions, and between the pre-RFT and post-RFT conditions. As we expected, jobless stress induced an incoherent and erratic heart rhythm pattern, together with low oscillation amplitude of heart rate (subject (b,d), pre-RFT). However, relative regular and coherent heart rhythm pattern, with a concomitant increase in oscillation amplitude, has been observed in the BL subjects (a,c) and post-RFT subjects (b,d) conditions, respectively, which implied that 3-week RFT biofeedback speed up the recovery of the cardiovascular system from the adverse impacts of jobless stress.

As shown in Table 5, quantitative analysis of the coherent level of heart rhythm pattern (CR) also demonstrated that 3-week RFT biofeedback associated with increased coherence in the heart's rhythmic activity. Compared with BL and post-RFT conditions, mean CR value in pre-RFT condition was much lower (p < 0.05), and there was no significant difference has been observed between BL and post-RFT conditions. Repeated measures ANOVA test also showed a significant changes of 3-week RFT biofeedback training with regard to coherent level of heart rhythm pattern (F(1, 33) = 4.664, p = 0.016), which indicated 3-week RFT biofeedback implemented by our biofeedback system has produced an apparent improvement in ANS synchronization and manifested a sine-wave-like oscillating mode of heart rhythm (positive emotion-related rhythm).

Evidence suggests that depressed HRV temporal measures can predict future adverse health outcomes, such as cardiac morbidity and mortality in high-risk populations [31]. Also, blunted HRV temporal measures may indicate a decreased ability for the heart to compensate or react appropriately during stressful or otherwise demanding situations [34]. Our studies confirmed that the subjects in unemployed group before 3-week RFT biofeedback training were associated with much lower HRV temporal measures. Compared with BL condition, the significant decreases in SDNN (p < 0.001) and rMSSD (p < 0.01) have been observed during the pre-RFT condition of unemployed group, the former reflected decreased overall HRV and the abilities of ANS modulation (approximately 1.99-fold larger in the post-RFT than the pre-RFT condition), the latter indicated the suppression of parasympathetic activation (approximately 1.49-fold larger in the post-RFT than the pre-RFT condition) under the jobless stress. However, after 3-week RFT biofeedback with our biofeedback system, the subjects in unemployed group have achieved significant increases in mean SDNN (p < 0.001) and rMSSD (p < 0.05), which indicated enhanced responsiveness of the cardiac autonomic system to psychophysiological stress. Furthermore, repeated measures ANOVA test also demonstrated that 3-week RFT biofeedback training have profound effects on HRV SDNN (F(1, 33) = 11.386, p < 0.001) and rMSSD (F(1, 33) = 4.982, p = 0.015) of unemployed subjects.

Chronic stress of the unemployed population is associated with medical problems because it sustains sympathetic nervous system (SNS) arousal with a general suppression of the parasympathetic nervous system (PNS), allowing the body's energy to focus on managing or escaping the stressor. Prolonged SNS activity however causes the body to become exhausted, weak and disease prone [35]. Investigators have shown that modulation of the sympathetic cardiac inputs contributes to the LF power and modulation of cardiac parasympathetic activity to the HF power [36]. And the LF/HF ratio has been used as an index to reflect the balance of sympatho-vagal tone. Therefore, in our study, compared to BL condition in the employed group, significantly increased HRV LF component (p < 0.01), and decreased HRV HF component (p < 0.01) during the pre-RFT condition in the unemployed group again confirmed these previous observations. Significant increases in LF/HF ratio (p < 0.001) also associated with less synchronization in the reciprocal action of the parasympathetic and sympathetic branches of the ANS in unemployed population (pre-RFT). However, after 3-week RFT biofeedback, significantly decreased HRV LF component (p < 0.01) and increased HRV HF component (p < 0.01) have been observed in the post-RFT condition relative to the pre-RFT condition, together with a concomitant decrease in LF/HF ratio (p < 0.01), suggesting that high-degree interaction between the activity of the SNS and PNS has been achieved by the unemployed subjects after 3-week RFT biofeedback, which allowed the unemployed subjects for more effective responses to demands and stressors, and helped them return to a stable, homeostatic state from adverse impact induced by jobless stress. This was also corroborated by the finding of a significant increase in the TP (p < 0.01) during the post-RFT condition (compared with pre-RFT), which indicated enhanced responsiveness of the cardiac autonomic system to psychophysiological stimuli in unemployed population after 3-week biofeedback training with our wearable biofeedback system.

Furthermore, as shown in Table 5, the SDNN and TP were approximately 1.99-fold (increased by 98.9%) and 1.97-fold (increased by 96.9%) larger in the post-RFT than the pre-RFT condition respectively, which indicated the ANS's regulating function and coping ability have doubled after 3-week RFT-based respiration training. In the frequency domain, the LF/HF ratio in the pre-RFT condition was approximately 3.34-foldand 2.93 larger than BL (employed group) and post-RFT (unemployed group) condition respectively, which implied the unemployed subjects have predominance in the SNS activation when under the jobless stress, and the balance between the SNS and PNS was achieved after 3-week RFT-based respiration training.

## 6. Conclusions

Wearable biofeedback is an emerging application field that combines modern biofeedback theories with state-of-the-art biosensor platform and BSN technologies. The multi-biosensor platform that we have constructed is universal and has low power consumption, low complexity, high compatibility, and high extendibility, which was used to create a RFT-based HRV biofeedback training device. Extensive bench and *in situ* testing results have shown that this biofeedback system worked as intended. The experimental study was conducted on wearable training patterns and resultant HRV. The results in all of the experiments suggested that this wearable biofeedback system with integrated RFT biofeedback strategy was appropriate for decreasing sympathetic arousal, increasing parasympathetic activity and enhancing overall capability of ANS modulation, which supporting the application of RFT biofeedback in the stress management and emotional control in daily life for unemployed population. We believe that the BSN platform and the wearable device could facilitate the research and development activities for ubiquitous and low-cost healthcare for the unemployed population.

Nevertheless, to address the limitations of our wearable biofeedback system, it still has the inherent disadvantage of obtrusiveness. Moreover, it is necessary for user to master a certain degree of professional knowledge during the RFT-based respiration training, which limited the usageof this wearable biofeedback system for self feedback and training of unemployed subjects in daily life. Therefore, in the future studies, we will find more efficient sensor modalities and combined manners (such as textile based sensors) for stress management of unemployed subjects in daily life, which should satisfy multiple requirements: minimized obtrusiveness, maximized the accuracy of stress assessment, and simplified subsequent complexity of RFT-based respiration training program. Furthermore, physiological researches have showed that PPG could be used as a surrogate of ECG during non-stationary condition [37], and respiration signal could be derived from a single-channel ECG signal (ECG derived respiration, EDR) [38]. Consequently, it is possible to use only one sensor (ECG sensor) for RFT-based biofeedback training, which basically needs two or more sensors in current release of our wearable biofeedback system. In the next study, we will prepare the upgrade to meet the simplicity and data fusion requirements of our wearable system through integrating the EDR algorithm into the software system and designing the information theoretic framework for multi-sensors' data fusion.

## Figures and Tables

**Table 1.** Specifications of sensor's amplifier and filter.

**Part name**	**Character**	**Gain**
Pre-amplifier (Instrument Amplifier)	Differential input impendence: 10 Gohm‖2 pF; CMRR: 120 dB; Input bias current: 1 nA; Input offset voltage: 25 μV	ECG: 20 dB PPG: 20 dB Respiration: 20 dB
High Pass Filter (LPF)	2nd-order Butterworth filter Cutoff frequency: ECG, 0.5 Hz; PPG, 0.1 Hz; Respiration: 0.1 Hz	0 dB
Low Pass Filter (HPF)	5th-order Bessel filter Cutoff frequency: ECG, 40 Hz; PPG, 20 Hz; Respiration: 10 Hz	0 dB
2rd Amplifier	Invert Amplifier	ECG:40 dB PPG: 20 dB Respiration:10 dB
Notch Filter	60 Hz Twin-T notch filter	0 dB

**Table 2.** HRV and respiration variables in our software system for stress assessment and biofeedback application [31].

**Variable**	**Unit**	**Description**

Selected Time Domain Measures of HRV (or PRV)		
SDNN	ms	Standard deviation of all RR intervals
rMSSD	ms	The square root of the mean of the sum of the squares of differences between adjacent RR intervals
Selected Frequency Domain Measures of HRV (or PRV)		

Total Power (TP)	ms^2^	The variance of RR intervals over the temporal segment (≤0.4 Hz)
LF norm	nu	Low frequency (LF, 0.04–0.15 Hz) power in normalized units
HF norm	nu	High frequency (HF, 0.15–0.4 Hz) power in normalized units
LF/HF	%	Ratio LF norm[nu]/HF norm[nu]
Selected Respiratory Measures		

Breathing Rate	b/m	Average breathing rate per minute in respiration recordings
Inspiration time	s	Relative time ofinspiration within respiration cycle
Expiration time	s	Relative time ofexpiration within respiration cycle

**Table 3.** Corresponding relation between the mean values of HRV measures and stress level [31].

**Variable**	**Mean Value**	**Stress Level**
SDNN	>50	Non-Stressed, ANS's regulating function and stress coping abilityis good
	35–50	Normal, ANS's regulating function and stress coping ability is normal
	20–35	High, there's risk of developing stress induced disease
	<20	Very High,there's high risk of having chronic stress induced disease

LF/HF	>2.0	Hyper Sympathetic activation, related to acute stress
	0.5–2.0	Normal,clinically considered as ANS balanced status
	<0.5	Hyper Parasympathetic activation, related to chronic stress

**Table 4.** Summary of the respiration measures.

	**Employed group**	**Unemployed group**		**ANOVA**	
Parameter	BL	pre-RFT	post-RFT	*F*	*P*
Breathing rate (b/m)	12.2 ± 0.6	19.3 ± 0.9 ***∧∧	13.5 ± 1.3 ∧∧	19.365	<0.001
Mean Exp. Time (s)	3.11 ± 0.48	1.04 ± 0.12 ***∧∧	2.67 ± 0.55 ∧∧***	15.886	<0.001
Mean Insp. Time (s)	1.81 ± 0.26	2.01 ± 0.40 *∧	1.78 ± 0.24 ∧	6.067	0.012

* , *p* < 0.05;

** , *p* < 0.01;

*** , *p* < 0.001, BL *vs.* pre-RFT or post-RFT;

∧ , *p* < 0.05;

∧∧ , *p* < 0.01;

∧∧∧ , *p* < 0.001, pre-RFT *vs.* post-RFT.

**Table 5.** Mean values and statistics of measured HRV and CR.

**Parameter**	**Employed group**	**Unemployed group**		**ANOVA**	

	**BL**	**pre-RFT**	**post-RFT**	**F**	**P**
CR	1.98 ± 0.45	0.87 ± 0.21 *∧	1.74 ± 0.32 ∧	4.664	<0.05
SDNN	56.4 ± 5.3	27.8 ± 3.8 ***∧∧∧	55.3 ± 6.1 ∧∧∧	11.386	<0.001
rMSSD	30.3 ± 1.3	19.4 ± 3.6 **∧	28.9 ± 7.3 ∧	4.982	<0.05
LF	68.7 ± 8.2	86.5 ± 10.2 **∧∧	65.7 ± 7.6 ∧∧	10.854	<0.01
HF	31.3 ± 8.2	13.5 ± 4.2 **∧∧	34.3 ± 7.6 ∧∧	8.046	<0.01
LF/HF	2.19 ± 0.46	6.41 ± 1.52 ***∧∧∧	1.92 ± 0.35 ∧∧∧	7.024	<0.01
TP	2154 ± 446.2	998 ± 254.7 **∧∧	1,966 ± 689.1 ∧∧	8.546	<0.01

* , *p* < 0.05;

** , *p* < 0.01;

*** , *p* < 0.001, BL *vs.* pre-RFT or post-RFT;

∧ , *p* < 0.05;

∧∧ , *p* < 0.01;

∧∧∧ , *p* < 0.001, pre-RFT *vs.* post-RFT.
